# Light Requirement Dynamics in Three Common Submerged Macrophytes: From Establishment to Peak Biomass

**DOI:** 10.3390/plants15071066

**Published:** 2026-03-31

**Authors:** Mengmei Liu, Mansen Liu, Yan Li, Kazi Belal Uddin, Yongjing Zhao

**Affiliations:** 1 State Key Laboratory of Lake and Watershed Science for Water Security, Institute of Hydrobiology, Chinese Academy of Sciences, Wuhan 430072, China; liumengmei@ihb.ac.cn (M.L.); liums@ihb.ac.cn (M.L.); belal_bau@yahoo.com (K.B.U.); zhaoyj@ihb.ac.cn (Y.Z.); 2 University of Chinese Academy of Sciences, Beijing 100049, China

**Keywords:** light–biomass model, light compensation point, *Vallisneria natans*, *Hydrilla verticillata*, *Myriophyllum spicatum*, ecological restoration

## Abstract

The deterioration of the underwater light environment is a key driver of submerged vegetation decline in shallow lakes. However, previous studies have largely focused on the short-term light needs of plants, failing to capture their dynamic requirements across the entire growth period. To quantify these stage-specific dynamics, we investigated three common submerged macrophytes: the rosette-type *Vallisneria natans*, erect-type *Hydrilla verticillata*, and canopy-forming *Myriophyllum spicatum*. Using mesocosm enclosures, we established eight bottom light gradients (0–20% of ambient light intensity) during both seedling and rapid growth stages to assess growth responses. Key findings are as follows: (1) Light requirements varied by species: *V. natans* < *H. verticillata* < *M. spicatum*. (2) Growth traits exhibited distinct responses: shoot density and biomass increased progressively with light, while plant height showed a unimodal response (increasing then declining), reflecting a shift in energy allocation. (3) Light requirements increased with developmental stage: the light compensation point for *V. natans*, *H. verticillata*, and *M. spicatum* increased from 2.1%, 4.4%, and 4.7% (seedling stage) to 3.3%, 10.5%, and 24.1% (rapid growth stage), respectively. (4) An integrated light–biomass model showed that achieving specific biomass targets required 2.4 to 4.7 times more light during rapid growth than during the seedling stage. This study quantifies stage-specific light requirements for submerged macrophytes, providing a theoretical basis for vegetation restoration and light management in shallow lakes.

## 1. Introduction

Submerged macrophytes are core structural and functional components of shallow lake ecosystems, playing indispensable roles in purifying water, stabilizing substrates, providing habitats, and maintaining biodiversity [1,2]. However, increasing global eutrophication has fueled widespread phytoplankton proliferation, resulting in a sustained decline in water transparency and consequently deteriorating underwater light conditions, becoming a key driver of the widespread decline or loss of submerged vegetation in many lakes [3,4]. Light is the sole energy source for photosynthesis in submerged plants, and its intensity and availability directly regulate their survival, growth, reproduction, and community distribution [5,6].

A critical but often overlooked aspect is that plant light requirements are not static but vary dynamically across different developmental stages [7,8]. While extensive research has been conducted on the light requirements of submerged macrophytes, most studies have focused on short-term, stage-specific responses (e.g., seedling stage) or organ-level physiological measurements (e.g., leaf photosynthetic rates) [9,10,11,12]. These approaches, although valuable, fail to capture how light demands change as plants grow from establishment to peak biomass. Moreover, a predictive framework that integrates stage-specific light requirements into a whole-growth-period model remains lacking. As a result, it is difficult for restoration practitioners to determine the dynamic light conditions needed to achieve specific biomass targets at different growth stages.

Therefore, the primary objective of this study was to quantify the dynamic light requirements of submerged macrophytes across their key growth phases and to develop an integrated light–biomass model for the entire growth period. We selected three common species representing different life forms in the shallow lakes of the middle and lower Yangtze River basin: the rosette-type *Vallisneria natans*, the erect-type *Hydrilla verticillata*, and the canopy-forming *Myriophyllum spicatum*. These species also differ in nutrient uptake strategies. While *V. natans* relies primarily on root uptake from sediments [13], *H. verticillata* and *M. spicatum* can absorb nutrients through both roots and shoots [14,15]. This physiological distinction may influence their responses to light limitation.

We hypothesized that light requirements increase with developmental stage for all species. To test this hypothesis, we conducted light-controlled mesocosm experiments during both the seedling (spring) and rapid growth (summer) stages. Using canopy-top light intensity as the key variable, we: (1) established stage-specific light–growth rate relationships; (2) derived the corresponding light compensation points (LCPs); and (3) integrated these stage-specific relationships into a whole-growth-period biomass accumulation model. This framework allows us to estimate the dynamic light requirements needed to achieve predefined biomass targets, offering a practical tool for guiding lake restoration and light management.

## 2. Results and Discussion

### 2.1. Light Conditions

During the seedling stage, natural light intensity (*l*_A_) ranged from 1091 to 1265 μmol/m^2^/s across treatments, while bottom light intensity (*l*_Z_) increased progressively from 1 to 276 μmol/m^2^/s as shading decreased, yielding the ratio of *l*_Z_ to *l*_A_ (*L*_B_) ranging from 0.1% to 24.9%. During the rapid growth stage, ambient light intensity was higher (*l*_A_: 1545–1795 μmol/m^2^/s), and *l*_Z_ ranged from 3 to 477 μmol/m^2^/s, with actual *L*_B_ values between 0.2% and 27.8% (Table 1). Although the measured *L*_B_ deviated slightly from the target levels, the intended light gradient was successfully maintained across treatments, providing a reliable basis for assessing plant growth responses.

### 2.2. Plant Growth Performance

**Figure 1:**
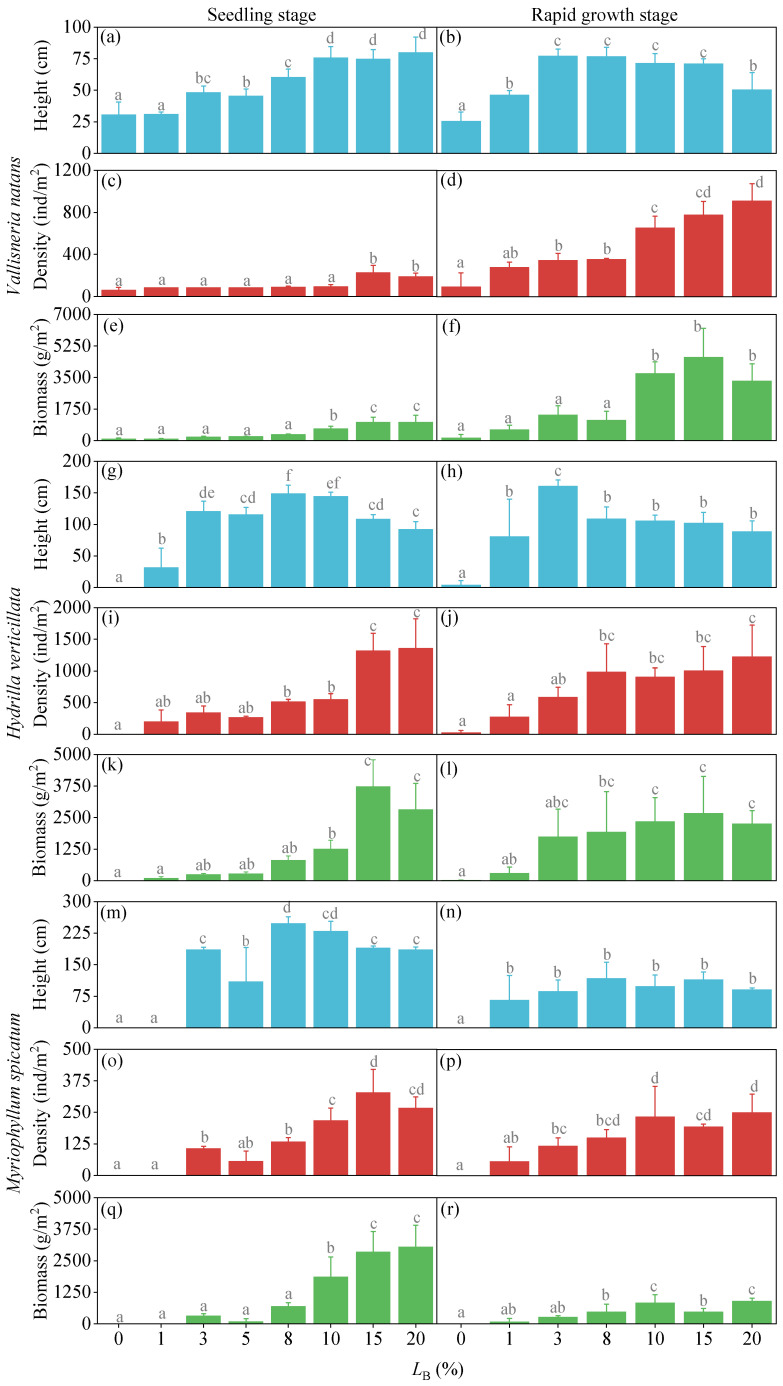
Plant height, shoot density, and biomass of *Vallisneria natans*, *Hydrilla verticillata*, and *Myriophyllum spicatum* under different light treatments following the seedling and rapid growth stage experiments (mean ± SD). (**a**–**f**) *V. natans*; (**g**–**l**) *H. verticillata*; (**m**–**r**) *M. spicatum*–height, density, and biomass at both the seedling and rapid growth stages, respectively. *L*_B_: Ratio of bottom light intensity to natural light intensity. Within each panel, bars not sharing a common lowercase letter are significantly different (*p* < 0.05).

For *V. natans* during the seedling stage, all measured traits increased with bottom light availability. Plant height was the most responsive, rising from 31 cm under the lowest light (0–1%) to 75–80 cm under the highest light (10–20%) (Figure 1a). Increases in shoot density and biomass were more gradual. Both parameters remained low and statistically similar across the 0–8% light treatments (density: 61–89 ind/m^2^; biomass: 82–332 g/m^2^). Above 8% light, density and biomass increased slightly, reaching 189–228 ind/m^2^ and 1017–1026 g/m^2^, respectively, in the 15–20% light groups (Figure 1c,e). During the rapid growth stage, shoot density and biomass continued to increase with light availability. Density rose from 94 to 278 ind/m^2^ at 0–1% light to 778–911 ind/m^2^ at 15–20% light (Figure 1d). Biomass was low (135–1421 g/m^2^) up to 8% light, but increased markedly to 3298–4620 g/m^2^ at 10–20% light (Figure 1f). In contrast, plant height exhibited a unimodal response: it was lowest (25 cm) under 0% light, peaked at 71–77 cm within the 3–15% light range, and then declined to 50 cm at 20% light (Figure 1b).

For *H. verticillata*, shoot density and biomass increased with light availability in both growth stages, whereas plant height exhibited a unimodal response, rising initially before declining at higher light levels. During the seedling stage, plant height was absent in the 0% light treatment, peaked at 145–149 cm under 8–10% light, and decreased to 92–109 cm in the 15–20% light groups (Figure 1g). Both density and biomass were zero in the 0% treatment, increasing to 1317–1356 ind/m^2^ and 2806–3724 g/m^2^, respectively, under 15–20% light (Figure 1i,k). In the rapid growth stage, plant height was minimal (4 cm) in the 0% treatment, reached a maximum of 161 cm at 3% light, and then declined to 88–109 cm across the 8–20% light range (Figure 1h). Shoot density remained low (22–278 ind/m^2^) under 0–1% light but rose to 906–1222 ind/m^2^ at 8–20% light (Figure 1j). Biomass increased sharply from only 6 g/m^2^ in the 0% treatment to 1742–2670 g/m^2^ across the 3–20% light gradient (Figure 1l).

For *M. spicatum* during the seedling stage, shoot density and biomass increased with greater light availability, while plant height exhibited a unimodal response. Plant height was absent in the 0–1% light treatments, peaked at 229–248 cm under 8–10% light, and then declined to 185–189 cm in the 15–20% light groups (Figure 1m). Shoot density also showed no survival in the 0–1% treatments, rising to 267–328 ind/m^2^ at 15–20% light (Figure 1o). Biomass remained low (0–685 g/m^2^) up to 8% light, but increased substantially to 2852–3054 g/m^2^ in the 15–20% light treatments (Figure 1q). During the rapid growth stage, all growth metrics of *M. spicatum* increased with light availability. Plant height was absent in the 0% treatment, but reached 66–117 cm across the 1–20% light range, with little variation among these groups (Figure 1n). Shoot density was low (0–56 ind/m^2^) under 0–1% light and increased to 50–250 ind/m^2^ in the 8–20% light treatments (Figure 1p). Biomass remained low (0–257 g/m^2^) up to 3% light, rising to 903 g/m^2^ at the highest light level (20%) (Figure 1r).

The above analysis reveals pronounced differences in light response among species, among growth metrics within species, and between developmental stages. Interspecific comparisons indicated a clear shade tolerance hierarchy. The rosette-type *V. natans* was the most shade-tolerant, surviving even in the 0% light treatment. In contrast, the erect-type *H. verticillata* (dead in the 0% treatment) and the canopy-forming *M. spicatum* (dead in the 0–1% treatments) exhibited progressively higher minimum light requirements. This pattern can be attributed to the combined effects of morphological structure, nutrient uptake strategies, and energy allocation patterns among the three species [16,17]. Notably, the survival of *V. natans* under complete darkness can be facilitated by its well-developed belowground tissues, which serve as reserves for carbohydrates that sustain basal metabolism during prolonged light deprivation [6,18]. Comparison among growth metrics showed that shoot density and biomass of all three species increased continuously and significantly with light availability in both stages, suggesting that none reached light saturation within the experimental range. In contrast, plant height consistently exhibited a unimodal response—increasing initially before declining at higher light levels. This pattern reflects a dynamic energy allocation strategy in heterogeneous light environments: under low light (0–8%), plants prioritize stem elongation to compete for light; when light becomes sufficient (10–20%), resources are reallocated from vertical growth to tillering and biomass accumulation, resulting in reduced height growth. Comparison between growth stages highlighted greater vulnerability to low bottom light during the seedling stage. At this stage, *V. natans* grew very slowly in the 0–8% treatments, *H. verticillata* died in the 0% treatment, and *M. spicatum* died in the 0–1% treatments. During the rapid-growth stage, taller plants could access light higher in the water column, reducing their dependence on bottom light. Consequently, both *V. natans* and *H. verticillata* survived in the 0% treatment, while only *M. spicatum* died under complete shading.

### 2.3. Light Intensity Emerges as the Dominant Driver of Absolute Growth Rate of Biomass (AGR)

A wide gradient of light levels was successfully established across treatments (Table 1), whereas other key environmental factors (e.g., water temperature (WT), total nitrogen (TN)) exhibited only minimal fluctuations and showed no significant inter-group differences (Table S1). Pearson correlation analysis between *AGR* and environmental factors (Table 2) revealed that, across all species and growth stages, the ratio of light intensity measured at the plant top to the ambient light just above the shade nets (*L*_T_) exhibited the strongest correlations with *AGR*, with correlation coefficients ranging from 0.75 to 0.93 (all *p* < 0.05). In contrast, correlations with WT, pH, and nutrients were consistently lower, with most |r| values below 0.7 and several non-significant.

The significant positive correlations between *AGR* and both WT and pH can be attributed to collinearity with light intensity. Treatments receiving higher light intensity experienced slightly elevated WT, and the enhanced photosynthetic activity under high light conditions consumed more dissolved CO_2_, leading to increased pH values. Throughout the experiment, TN and total phosphorus (TP) concentrations remained below levels known to limit plant growth [19,20]. The observed negative correlations between *AGR* and TN/TP are therefore best explained as a consequence of plant uptake, higher growth rates resulted in greater nutrient assimilation from the water column, reducing residual nutrient concentrations.

Collectively, these analyses demonstrate that while WT and pH covaried with light intensity, and nutrient concentrations reflected plant uptake rather than growth limitation, light intensity was the primary environmental factor driving *AGR*. The strong, consistent correlations between *AGR* and *L*_T_ across all species and stages, combined with the absence of systematic variation in other factors among treatments, confirm that light intensity was the dominant regulator of growth in this experiment.

### 2.4. Light–Growth Rate Models and Derivation of Light Compensation Point (LCP) and Light Saturation Point (LSP) for Each Growth Stage

**Figure 2:**
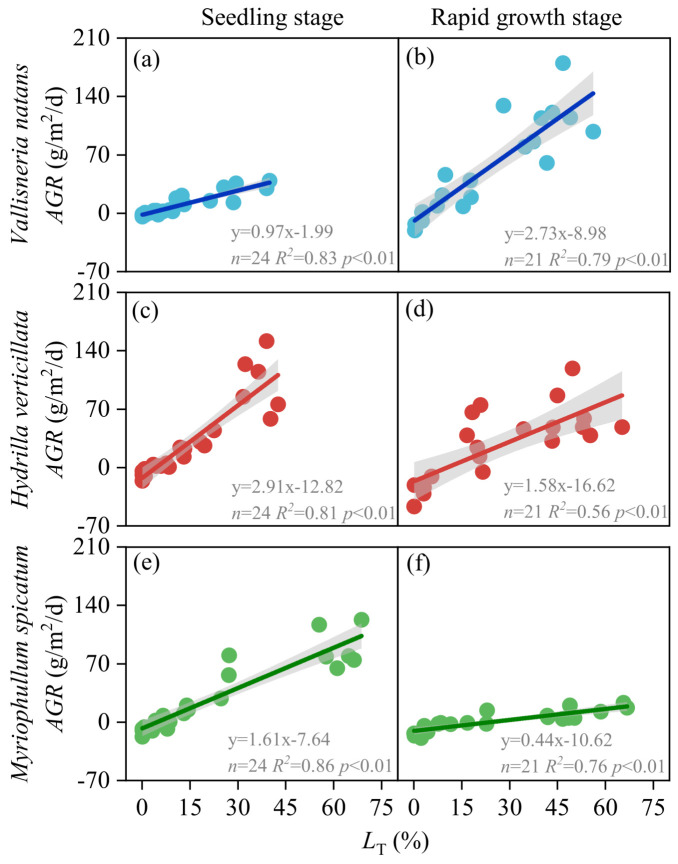
Relationships between the absolute growth rate (*AGR*) and the ratio of canopy-top light intensity to natural light intensity (*L*_T_) for *Vallisneria natans*, *Hydrilla verticillata*, and *Myriophyllum spicatum* during the seedling and rapid growth stages. (**a**,**b**) *V. natans*; (**c**,**d**) *H. verticillata*; (**e**,**f**) *M. spicatum*–*AGR* as a function of *L*_T_ at the seedling and rapid growth stages, respectively. Scatter points indicate raw data; solid lines represent linear regression fits; shaded areas denote 95% confidence intervals. Regression parameters (equation, *n*, *R^2^*, *p*) are provided in each subplot.

Using *L*_T_ as the predictor, light–growth rate relationships were constructed for *V. natans*, *H. verticillata*, and *M. spicatum* during the seedling and rapid growth stages (Figure 2). It was found that the rate of increase in growth rate with light for *V. natans* was lower in the seedling stage than in the rapid growth stage, while for *H. verticillata* and *M. spicatum*, it was higher in the seedling stage. The light–growth rate models for each stage of the three species are as follows:

- *V. natans*

Seedling stage:           *AGR*_Vn_S_ = 0.97*L*_T_Vn_S_ − 1.99 (1)

Rapid growth stage:      *AGR*_Vn_R_ = 2.73*L*_T_Vn_R_ − 8.98 (2)

- *H. verticillata*

Seedling stage:           *AGR*_Hv_S_ = 2.91*L*_T_Hv_S_ − 12.82 (3)

Rapid growth stage:      *AGR*_Hv_R_ = 1.58*L*_T_Hv_R_ − 16.62 (4)

- *M. spicatum*

Seedling stage:           *AGR*_Ms_S_ = 1.61*L*_T_Ms_S_ − 7.64 (5)

Rapid growth stage:      *AGR*_Ms_R_ = 0.44*L*_T_Ms_R_ − 10.62 (6)

where *AGR*_Xx_S_ and *AGR*_Xx_R_ represent the absolute growth rates (g/m^2^/d) of species X during the seedling (S) and rapid growth (R) stages, respectively, with subscripts ‘Vn’, ‘Hv’, and ‘Ms’ denoting *V. natans*, *H. verticillata*, and *M. spicatum*. Similarly, *L*_T_Xx_S_ and *L*_T_Xx_R_ denote the ratio of canopy-top to natural light intensity (%) for the corresponding species and growth stage.

Based on the stage-specific light–growth rate models (Equations (1)–(6)), the LCP and LSP were determined for each growth stage of the three submerged macrophytes. The LCP for *V. natans* was 2.1% of ambient light (mean canopy-top light: 24 μmol/m^2^/s; range: 2–40) in the seedling stage and 3.3% (55 μmol/m^2^/s; 9–73) in the rapid growth stage. For *H. verticillata*, the seedling and rapid growth stage LCPs were 4.4% (52 μmol/m^2^/s; 3–83) and 10.5% (174 μmol/m^2^/s; 27–232), respectively. For *M. spicatum*, the corresponding LCPs were 4.7% (55 μmol/m^2^/s; 3–89) and 24.1% (400 μmol/m^2^/s; 63–532). Within the experimental light gradient, light saturation was not reached for any species. Therefore, the LSP for each stage is inferred to be greater than the highest tested light level: for *V. natans*, >39.8% (467 μmol/m^2^/s; 27–751) in the seedling stage and >56.0% (930 μmol/m^2^/s; 146–1237) in the rapid growth stage; for *H. verticillata*, >42.6% (500 μmol/m^2^/s; 29–804) and >65.2% (1083 μmol/m^2^/s; 170–1440); and for *M. spicatum*, >68.7% (807 μmol/m^2^/s; 46–1297) and >66.8% (1109 μmol/m^2^/s; 175–1475).

Comparison of LCPs within the same species revealed a consistent increase from the seedling stage to the rapid growth stage, a finding rarely documented in prior studies on submerged macrophyte light requirements. A possible mechanism is that as plants develop, the proportion of non-photosynthetic (e.g., roots, rhizomes, reproductive structures) and weakly photosynthetic tissues (e.g., stems) increases (from <20% in seedlings to 30–40% during rapid growth) [13,21,22], thereby elevating the whole-plant light requirement to support growth and reproductive demands. Across species, LCPs followed the order *V. natans* < *H. verticillata* < *M. spicatum*, which aligns with earlier reports and can be attributed to differences in physiological traits and niche differentiation among these species [23,24].

The stage-specific LCP and LSP values derived from our models were compared with literature data obtained via microcosm (organ-level) and mesocosm (whole-plant) experiments (Table 3). For *V. natans*, only the seedling-stage LCP from microcosm studies (4.3–9.4 μmol/m^2^/s) was comparable to our model-derived value. Other reported values, including rapid-growth-stage LCPs and all LSPs from both microcosm and mesocosm experiments, were lower than our estimates. For *H. verticillata*, the seedling-stage LCP from microcosm studies (15.8–28.9 μmol/m^2^/s) and the rapid-growth-stage LCP from one mesocosm study (235–303 μmol/m^2^/s) aligned with our model results. Other literature values were lower. For *M. spicatum*, the seedling-stage LCP from microcosm studies (21.6–51.0 μmol/m^2^/s) matched our model output, while other reported LCP and LSP values were lower. In summary, microcosm-derived LCPs aligned with our model only during the seedling stage. This is likely because seedling growth is predominantly leaf-based, similar to the isolated leaves or apical shoots used in microcosm assays. In later stages, energy demands increase for processes like stem elongation, clonal propagation, and reproduction; thus, organ-level instantaneous measurements fail to capture the whole-plant’s integrated light requirement. Discrepancies with prior mesocosm studies can be attributed to methodological differences. Earlier work estimated critical light levels based on plant survival or growth thresholds across discrete light gradients. In contrast, our approach establishes a continuous linear relationship between light intensity and growth rate, allowing for a more precise and mechanistically grounded estimation of LCP and LSP across a continuous parameter space.

### 2.5. Integrated Light–Biomass Model for Entire Growth Period

We constructed biomass increment models for each stage from the stage-specific light–growth rate models and summed them to model maximum biomass over the entire growth period, which we define as spanning from establishment to peak biomass.

- *V. natans*

Seedling stage:        Δ*B*_Vn_S_ = *AGR*_Vn_S_*T*_S_ = (0.97*L*_T_Vn_S_ − 1.99)*T*_S_ (7)

Rapid growth stage:   Δ*B*_Vn_R_ = *AGR*_Vn_R_*T*_R_ = (2.73*L*_T_Vn_R_ − 8.98)*T*_R_ (8)

Entire growth period:         *B*_Vn_Max_ = Δ*B*_Vn_S_ + Δ*B*_Vn_R_ (9)

- *H. verticillata*

Seedling stage:       Δ*B*_Hv_S_ = *AGR*_Hv_S_*T*_S_ = (2.91*L*_T_Hv_S_ − 12.82)*T*_S_ (10)

Rapid growth stage:  Δ*B*_Hv_R_ = *AGR*_Hv_R_*T*_R_ = (1.58*L*_T_Hv_R_ − 16.62)*T*_R_ (11)

Entire growth period:         *B*_Hv_Max_ = Δ*B*_Hv_S_ + Δ*B*_Hv_R_ (12)

- *M. spicatum*

Seedling stage:      Δ*B*_Ms_S_ = *AGR*_Ms_S_*T*_S_ = (1.61*L*_T_Ms_S_ − 7.64)*T*_S_ (13)

Rapid growth stage: Δ*B*_Ms_R_ = *AGR*_Ms_R_*T*_R_ = (0.44*L*_T_Ms_R_ − 10.62)*T*_R_ (14)

Entire growth period:         *B*_Ms_Max_ = Δ*B*_Ms_S_ + Δ*B*_Ms_R_ (15)

where Δ*B*_Vn_S_, Δ*B*_Hv_S_, Δ*B*_Ms_S_: Seedling-stage biomass increment (g/m^2^) for *V. natans*, *H. verticillata*, and *M. spicatum*, respectively; Δ*B*_Vn_R_, Δ*B*_Hv_R_, Δ*B*_Ms_R_: Rapid-growth-stage biomass increment (g/m^2^) for the three species; *B*_Vn_Max_, *B*_Hv_Max_, *B*_Ms_Max_: Maximum biomass (g/m^2^) over the entire growth period for the three species; *T*_S_, *T*_R_: Duration (days) of the seedling and rapid growth stages, respectively. Other variables are as defined in Equations (1)–(6).

### 2.6. Dynamic Light Demand Under Specific Biomass Targets

The required light intensity for each growth stage was calculated through a three-step process. First, a maximum biomass target was defined. Then, based on plant growth patterns, biomass increments were allocated to each stage (Equations (9), (12) and (15)). Finally, using light–biomass increment models (Equations (7), (8), (10), (11), (13) and (14)), the required light intensity for each stage was calculated.

In Table 4, this framework was applied by setting the maximum biomass targets to the average summer biomass of *V. natans*, *H. verticillata*, and *M. spicatum* recorded in Lakes Lu, Liangzi, Bao’an, and Niushan between 2001 and 2003. The results demonstrate a rapid increase in light demand with plant development: the light required during the rapid growth stage was 2.5, 2.4, and 4.7 times higher than during the seedling stage for *V. natans*, *H. verticillata*, and *M. spicatum*, respectively. Therefore, this model provides a practical tool for managers: by inputting a target restoration biomass, the necessary light conditions for each growth stage can be defined.

## 3. Materials and Methods

### 3.1. Study Area and Experimental System

**Figure 3:**
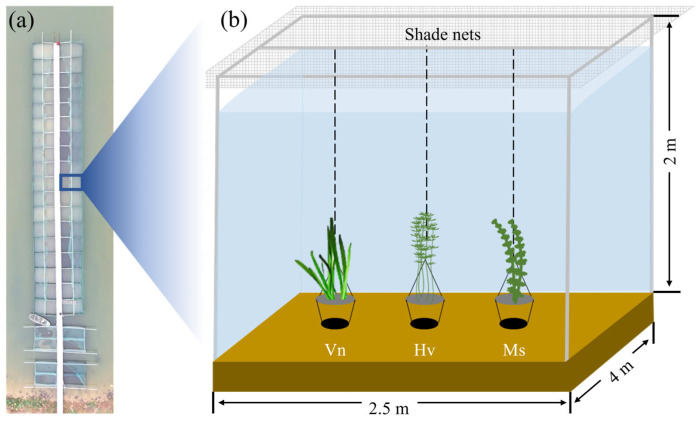
The experimental system. (**a**) Top view of the enclosure system; (**b**) Schematic diagram of the experimental system. Vn, *Vallisneria natans*; Hv, *Hydrilla verticillata*; Ms, *Myriophyllum spicatum*.

This study was conducted at the Bao’an Lake Experimental Limnological Research Station in Daye City, Hubei Province. The experimental enclosure system (Figure 3a) was set up in a pond (area approx. 3500 m^2^). Each enclosure measured 4 m × 2.5 m × 2 m (L × W × H; Figure 3b). During the experiment, the water depth in the enclosures was approximately 1.7 m.

### 3.2. Study Species

Three common submerged macrophytes were selected: *V*. *natans* (rosette-type), *H*. *verticillata* (erect-type), and *M*. *spicatum* (canopy-forming). Functional classification of the three species was based on growth morphology observed during the experimental period. Under our experimental conditions, *H. verticillata* biomass was uniformly distributed throughout the water column and did not form a distinct surface canopy. In contrast, *M. spicatum* biomass was concentrated in the upper portion of the plants, forming a distinct canopy at the water surface. Accordingly, *M. spicatum* was classified as canopy-forming and *H. verticillata* as erect-type. This classification is consistent with previous studies that have characterized *H. verticillata* as an erect-type species [33,34].

### 3.3. Experimental Setup

Shade nets of different densities were placed above the enclosures to create eight light gradients. *L*_B_ were 0%, 1%, 3%, 5%, 8%, 10%, 15%, and 20%. Each treatment had three replicates, utilizing a total of 24 enclosures. Three plastic pots were suspended in each enclosure for planting *V. natans*, *H. verticillata*, and *M. spicatum*, respectively. Experimental plants were collected from ponds near the Bao’an Lake Research Station, and the sediment in the plastic pots was taken from the corresponding enclosure.

The seedling-stage experiment was conducted from 5 May to 5 June 2023 (31 days). Seven individuals of each species (*V. natans*, *H. verticillata*, and *M. spicatum*) were planted, with one species per pot, in each enclosure. The pots were suspended 50 cm below the water surface for a one-week pre-cultivation period. Subsequently, five uniformly sized plants per species were retained for the formal experiment. At this time, the average plant heights for *V. natans*, *H. verticillata*, and *M. spicatum* were 18 cm, 18 cm, and 36 cm, respectively. Initial environmental parameters were WT 20.0–20.5 °C, pH 8.0–8.6, TN 0.61–0.95 mg/L, TP 0.01–0.02 mg/L, Chl *a* 1.2–8.3 µg/L. The rapid-growth-stage experiment was conducted from July 7 to August 8, 2022 (32 days), following the same planting and pre-cultivation procedures as described above. At the start of the experiment, the average plant heights for *V. natans*, *H. verticillata*, and *M. spicatum* were 37 cm, 33 cm, and 47 cm, respectively. Initial environmental parameters were WT 30.2–30.6 °C, pH 7.7–8.5, TP 0.02–0.05 mg/L, phytoplankton Chl *a* 1.5–18.4 µg/L. Basic environmental parameters for each treatment during both experimental periods are shown in Table S1.

### 3.4. Sampling and Measurement

Physical water parameters (such as depth, temperature, and light intensity) and plant growth (shoot number, plant height, and fresh weight) were monitored weekly. Here, light intensity refers to photosynthetically active radiation (PAR, 400–700 nm). Water chemistry, including TN, TP, and phytoplankton Chl *a*, was analyzed at the beginning and end of the experiment.

Water depth was measured with a portable ultrasonic depth sounder (SM-5, Speedtech, Sterling, VA, USA). Light intensity was measured using a light meter (LI-250A, LI-COR, Lincoln, NE, USA) between 11:30 and 12:30 in the following positions: above and below the shade net in air, at the air–water interface, and at 0.5 m, 1 m below the water surface, and 5 cm above the sediment. WT, pH, etc., were measured *in situ* using a multi-parameter probe (YSI ProPlus, YSI, Yellow Springs, OH, USA) at three depths: upper (0.5 m below surface), middle (mid-depth), and lower (0.5 m above sediment). Water samples were collected from the same three layers, then mixed, stored in 1 L bottles, and transported to the laboratory for the analysis of TN, TP, and phytoplankton Chl *a* according to standard methods [35]. At planting, an additional 50 randomly selected individuals per species, similar to those planted, were retained and weighed to determine the average initial fresh weight per plant. The initial biomass per pot was then estimated by multiplying this average fresh weight by the initial number of plants per pot. During each weekly monitoring, pots were slowly retrieved. The number of shoots per pot was counted. Subsequently, the heights of the three tallest plants were measured; if fewer than three plants were present, all plants were measured. At the end of the experiment, all plants were carefully removed, rinsed, blotted dry, and weighed to determine the final fresh biomass.

### 3.5. Data Analysis

Data were processed and analyzed using Microsoft Excel 2019, OriginPro 2021, and SPSS 26. The normality and homogeneity of variances of the data were verified prior to analysis. Differences in growth indicators among treatment groups were assessed by one-way analysis of variance (ANOVA), followed by Tukey’s honest significant difference (HSD) post hoc test for multiple comparisons where significant effects were found.

The absolute growth rate of biomass (*AGR*, g/m^2^/d) was calculated as:

*AGR* = (*B*_2_ − *B*_1_)/Δ*t* (16)

where *B*_1_ and *B*_2_ are the biomass (g/m^2^) at the beginning and end of the experiment, respectively, and Δ*t* is the experiment duration (d).

**Figure 4:**
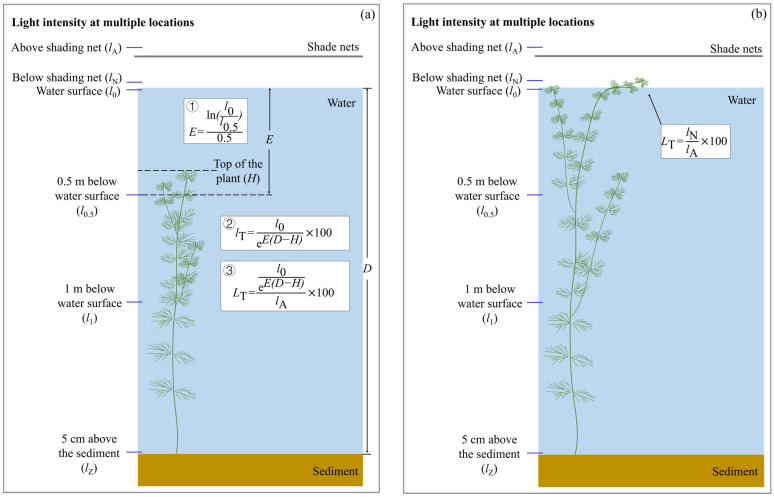
Schematic for calculating the ratio of light intensity at the plant top (100% of plant height) to ambient light intensity (*L*_T_). (**a**) When plant canopies are below the water surface; (**b**) When canopies spread horizontally at the water surface. *E* is the light attenuation coefficient, /m; *l*_0_ is the light intensity at the air–water interface, μmol/m^2^/s; *l*_0.5_ is the light intensity at 0.5 m below the water surface, μmol/m^2^/s; *l*_T_ is the plant apical light intensity, μmol/m^2^/s; e is the natural logarithm; *D* is the water depth, m; *H* is the plant height, m; *l*_A_ is the light intensity in the air above the shade nets, μmol/m^2^/s; *l*_N_ is the light intensity in the air below the shade nets, μmol/m^2^/s.

The light intensity at the plant top was used as the key variable to construct light–growth rate models. This approach was justified because photosynthetic tissues are primarily concentrated in the upper plant strata, where self-shading is minimal. Canopy-top light intensity was derived from vertical light profiles measured in the water column. Light attenuation coefficients were calculated separately for three depth intervals: (1) from the air–water interface to 0.5 m depth, (2) from 0.5 m to 1 m depth, and (3) from 1 m depth to 5 cm above the sediment. These coefficients were then applied to calculate the canopy-top light intensity for each plant and its corresponding ratio to ambient light intensity (*L*_T_). The complete calculation workflow is provided in Figure 4.

To evaluate the relationships between plant growth and environmental conditions, Pearson correlation analysis was performed between *AGR* and key environmental variables, including *L*_T_, WT, pH, TN, TP, and Chl *a*. The analysis was conducted separately for each species (*V. natans*, *H. verticillata*, *M. spicatum*) and each growth stage (seedling and rapid growth). Correlation coefficients (r) and their significance levels (*p* < 0.05) were calculated using OriginPro 2021. Data normality was verified using the Shapiro–Wilk test prior to analysis.

LCP is the light intensity at which photosynthesis balances respiration, resulting in no net carbon gain [36]. Operationally, we define LCP as the light intensity corresponding to zero absolute growth rate. Similarly, LSP is the minimum light intensity required for maximum photosynthesis [37]. In our study, LSP represents the minimum light intensity at which the growth rate is maximized.

## 4. Conclusions

This study quantified the growth responses of three submerged macrophytes (*V. natans*, *H. verticillata*, and *M. spicatum*) to light intensity during seedling and rapid growth stages, and developed an integrated whole-growth-period light–biomass model. The main conclusions are:

1. Shade tolerance ranked *V. natans* > *H. verticillata* > *M. spicatum*: *V. natans* survived at 0% light; *H. verticillata* survived at 0% light only during rapid growth; and *M. spicatum* failed to survive at 0–1% light.
2. Growth traits responded differently to light: shoot density and biomass increased continuously without saturation, whereas plant height peaked under low light (0–8%) and then declined at higher light (10–20%) as energy allocation shifted toward tillering and biomass accumulation.
3. Light requirements increased with growth stage: Seedling-stage LCPs were 2.1% for *V. natans*, 4.4% for *H. verticillata*, and 4.7% for *M. spicatum*, rising to 3.3%, 10.5%, and 24.1%, respectively, during the rapid growth stage.
4. Based on the entire-growth-period light–biomass model, dynamic light requirements for specific biomass targets can be determined, revealing that light demand during the rapid growth stage is 2.4–4.7 times that of the seedling stage.

In summary, submerged macrophyte light demand is both species-specific and stage-dependent. Effective restoration should consider not only shade tolerance but also dynamic light needs throughout growth to guide precise light management.

## Figures and Tables

**Table 1:** Measured light conditions under eight target light treatments during both the seedling and rapid growth stages (mean ± SD).

Growth Stages	Light Parameters	Experimental Treatments, *L*_B_							
		0%	1%	3%	5%	8%	10%	15%	20%
Seedling	*l*_A_ (μmol/m^2^/s)	1121 ± 567	1099 ± 516	1182 ± 486	1180 ± 549	1252 ± 515	1265 ± 525	1205 ± 579	1091 ± 480
	*l*_Z_ (μmol/m^2^/s)	1 ± 1	8 ± 5	30 ± 14	36 ± 28	78 ± 47	105 ± 65	222 ± 142	276 ± 132
	Actual *L*_B_ (%)	0.1 ± 0.0	0.7 ± 0.1	2.6 ± 0.7	2.9 ± 1.0	6.1 ± 0.8	8.0 ± 1.0	17.2 ± 4.8	24.9 ± 1.1
Rapid growth	*l*_A_ (μmol/m^2^/s)	1795 ± 402	1699 ± 412	1655 ± 462	-	1785 ± 361	1624 ± 502	1545 ± 552	1606 ± 560
	*l*_Z_ (μmol/m^2^/s)	3 ± 1	29 ± 13	64 ± 42	-	160 ± 97	334 ± 184	340 ± 183	477 ± 277
	Actual *L*_B_ (%)	0.2 ± 0.0	1.7 ± 0.1	3.7 ± 0.5	-	10.5 ± 1.4	18.5 ± 1.2	21.5 ± 2.1	27.8 ± 3.8

Note: *L*_B_, the ratio of light intensity measured at the sediment surface to the ambient light just above the shade nets; *l*_A_, light intensity in the air above the shade nets; *l*_Z_, light intensity at 5 cm above the sediment.

**Table 2:** Pearson correlation coefficients between absolute growth rate (*AGR*) and major environmental factors for each species during the seedling and rapid growth stages.

Species	Growth Stages	*L*_T_	WT	pH	TN	TP	Chl *a*
*Vallisneria natans*	Seedling	0.91 *	0.40	0.67	−0.66 *	−0.04	0.26
	Rapid growth	0.89 *	0.76 *	0.81 *	0.36	−0.41	0.35
*Hydrilla verticillata*	Seedling	0.90 *	0.60 *	0.64 *	−0.70 *	−0.03	0.27
	Rapid growth	0.75 *	0.43 *	0.70 *	0.26	−0.52 *	0.42
*Myriophyllum spicatum*	Seedling	0.93 *	0.42 *	0.67 *	−0.71 *	−0.08	0.18
	Rapid growth	0.87 *	0.63 *	0.75 *	0.33	−0.39	0.50 *

Note: *L*_T_, the ratio of light intensity measured at the plant top to the ambient light just above the shade nets; WT, water temperature; TN, total nitrogen; TP, total phosphorus; Chl *a*, phytoplankton chlorophyll *a*; “*” indicates a significant difference at *p* < 0.05.

**Table 3:** Comparison of light compensation point (LCP) and light saturation point (LSP): literature-reported values versus model-derived values.

Species	Growth Stages	Parameters	Literature Values (Method)	Model-Derived Values (This Study)	Notes/Comparison
*Vallisneria natans*	Seedling	LCP	4.3–9.4 μmol/m^2^/s (Microcosm, leaf) [25,26,27]; 0 < LCP ≤ 1% *l*_A_ (Mesocosm) [28,29]	2.1% *l*_A_ (24 μmol/m^2^/s)	Microcosm (leaf) value comparable to model. Mesocosm range lower.
		LSP	55.6–200.0 μmol/m^2^/s (Microcosm, leaf) [25,26]; ≤15% *l*_A_ (Mesocosm) [11]	>39.8% *l*_A_ (>467 μmol/m^2^/s)	All literature values lower than model estimate.
	Rapid growth	LCP	6.3 μmol/m^2^/s (Microcosm, leaf) [27]; 116–144 μmol/m^2^/s (Mesocosm, water layer) [30]	3.3% *l*_A_ (55 μmol/m^2^/s)	Literature values differ substantially from model.
		LSP	-	>56.0% *l*_A_ (>930 μmol/m^2^/s)	-
*Hydrilla verticillata*	Seedling	LCP	15.8–28.9 μmol/m^2^/s (Microcosm, leaf/apical) [25,26,31]	4.4% *l*_A_ (52 μmol/m^2^/s)	Microcosm range comparable to model.
		LSP	97.1–500.0 μmol/m^2^/s (Microcosm, leaf/apical) [25,26,31]; 25.0 μmol/m^2^/s (Mesocosm) [32]	>42.6% *l*_A_ (>500 μmol/m^2^/s)	Literature values lower.
	Rapid growth	LCP	235–303 μmol/m^2^/s (Mesocosm, water layer) [30]	10.5% *l*_A_ (174 μmol/m^2^/s)	Mesocosm value comparable to model.
		LSP	-	>65.2% *l*_A_ (>1083 μmol/m^2^/s)	-
*Myriophyllum spicatum*	Seedling	LCP	21.6–51.0 μmol/m^2^/s (Microcosm, leaf/apical) [9,10,25,26,27]	4.7% *l*_A_ (55 μmol/m^2^/s)	Microcosm range comparable to model.
		LSP	134–1000 μmol/m^2^/s (Microcosm, leaf/apical) [9,25,26,31]; ≤15% *l*_A_ (Mesocosm) [11]	>68.7% *l*_A_ (>807 μmol/m^2^/s)	Literature values lower.
	Rapid growth	LCP	5% *l*_A_ (Mesocosm) [12]	24.1% *l*_A_ (400 μmol/m^2^/s)	Literature value lower than model.
		LSP	-	>66.8% *l*_A_ (>1109 μmol/m^2^/s)	-

Note: *l*_A_ represents ambient light intensity.

**Table 4:** Dynamic light demand for the three submerged macrophytes under specific biomass targets.

Species	Growth Stages	Maximum Biomass Targets (g/m^2^)	Biomass Increments (g/m^2^)	Growth Days (d)	Light Demand (*L*_T_, %)
*Vallisneria natans*	Seedling	2000	200	90	4.3
	Rapid growth		1800	90	10.6
*Hydrilla verticillata*	Seedling	700	300	90	5.6
	Rapid growth		400	90	13.3
*Myriophyllum spicatum*	Seedling	600	300	90	6.8
	Rapid growth		300	90	31.7

Note: *L*_T_ is the ratio of light intensity at the plant top to ambient light intensity.

## Data Availability

The original contributions presented in this study are included in the article. Further inquiries can be directed to the corresponding author.

## Supplementary Materials

The following supporting information can be downloaded at: https://www.mdpi.com/article/10.3390/plants15071066/s1, Table S1: Variations in basic environmental parameters during the experimental period (mean ± SD).
