# Supplementary material for: Light Requirement Dynamics in Three Common Submerged Macrophytes: From Establishment to Peak Biomass
Source: Plants (Basel). 2026 Mar 31;15(7):1066. doi: 10.3390/plants15071066 (PMC13074490; doi:10.3390/plants15071066)
Supplement: Supplementary file 1 [file plants-15-01066-s001.zip › plants-4176259-supplementary.pdf]

Table S1. Variations in basic environmental parameters during the experimental period (mean ± SD).

Growth stages	Environmental parameters	Experimental treatments, L_B							
		0%	1%	3%	5%	8%	10%	15%	20%
Seedling	WT (℃)	22.88±2.88	22.89±2.86	22.63±2.65	22.81±2.83	22.75±2.79	22.72±2.65	23.02±2.70	22.98±2.68
	pH	7.92±0.15	8.01±0.14	8.20±0.11	7.86±0.14	7.84±0.13	7.98±0.12	8.22±0.16	8.53±0.16
	TN (mg/L)	0.93±0.12	1.06±0.15	1.03±0.14	0.86±0.10	0.71±0.09	0.61±0.01	0.66±0.02	0.61±0.08
	TP (mg/L)	0.01±0.01	0.02±0.00	0.02±0.00	0.03±0.01	0.01±0.01	0.01±0.01	0.02±0.01	0.01±0.01
	Chl *a* (μg/L)	1.74±1.18	1.52±0.64	1.44±0.32	5.46±1.72	3.03±1.07	4.55±1.93	9.02±0.97	1.59±0.11
Rapid growth	WT (℃)	31.56±1.05	31.45±1.07	31.46±1.04	-	31.46±1.08	31.69±0.99	31.75±1.01	31.74±0.99
	pH	7.85±0.17	7.82±0.12	8.16±0.22	-	7.92±0.15	8.30±0.08	8.33±0.20	8.36±0.28
	TN (mg/L)	0.63±0.21	0.40±0.12	0.82±0.03	-	0.50±0.07	0.59±0.02	0.84±0.19	1.78±1.54
	TP (mg/L)	0.03±0.01	0.03±0.00	0.04±0.01	-	0.02±0.01	0.02±0.01	0.04±0.02	0.03±0.02
	Chl *a* (μg/L)	2.73±0.45	5.23±2.41	15.17±1.25	-	10.54±2.75	7.20±2.04	15.17±3.42	9.63±3.48

Note: L_B, the ratio of light intensity measured at the sediment surface to the ambient light just above the shade nets; WT, water temperature; TN, total nitrogen; TP, total phosphorus; Chl *a*, phytoplankton chlorophyll *a*; "-" indicates no data.
